# **The neuroimmune network in Alzheimer’s and Parkinson’s diseases: from mechanistic insights to biomarker**-**guided immunotherapies and clinical translation**

**DOI:** 10.1007/s10787-026-02363-7

**Published:** 2026-08-21

**Authors:** Mohamed N. Fawzy

**Affiliations:** Department of Pharmacology and Toxicology, Faculty of Pharmacy, Sinai University-Arish Branch, Arish, 45511 Egypt

**Keywords:** Neuroimmune network, NLRP3 inflammasome, SIGLEC10, CAR-T cells, Microglia, Biomarkers, NT‑0796

## Abstract

Neurodegenerative disorders such as Alzheimer’s (AD) and Parkinson’s (PD) have traditionally been examined from the perspectives of neurons or microglia, resulting in constrained therapeutic achievements. Recent findings endorse a cohesive neuroimmune framework in which central nervous system (CNS)-resident microglia, border-associated macrophages, clonally proliferated CD8^+^ T cells, and peripheral signaling centers (IL-20 family, gut-brain axis) perpetuate chronic maladaptive inflammation via feed-forward mechanisms. This review critically examines investigational immunotherapies aimed at this network: the CNS‑penetrant NLRP3 inhibitor NT-0796 (Phase 1b/2a) demonstrated preliminary biomarker reductions in axonal damage and T-cell activation in PD but remains unapproved and necessitates further confirmatory trials; the anti‑SIGLEC10 antibody ONC-841 improved microglial phagocytosis of Aβ and tau in preclinical studies but has yet to commence human trials; and CAR‑based platforms (CAR-T/NK) remain in the nascent preclinical phase, facing significant delivery and toxicity challenges. A three-part biomarker framework, encompassing target engagement (CSF IL-1β, caspase-1), pharmacodynamic responses (neurofilament light chain, ASC specks), and predictive endotyping (T-cell clonality, complement profiles), is proposed to facilitate patient stratification by neuroimmune endotype. None of these agents have received regulatory approval for neurodegenerative conditions; all findings are preliminary. Effective immunotherapy may ultimately necessitate multi-node, network-aware combinations (e.g., inflammasome inhibition coupled with Treg augmentation) rather than single-target suppression. Embracing this complexity offers a roadmap for future disease-modifying therapies, though rigorous clinical validation remains essential.

## Highlights

- Neuroimmune networks, not microglia alone, drive chronic neuroinflammation in neurodegeneration.
- Clonally expanded CD8^+^ T cells actively contribute to neuronal damage in neurodegeneration.
- First-in-class CNS-penetrant inhibitor NT-0796 of the NLRP3 protein shows biomarker responses in Parkinson’s disease.
- Peripheral IL-20 family and gut-brain axis offer accessible targets for neuroimmune modulation.
- Biomarker-guided stratification and multi-node combinations are essential for neuroimmune therapy success.

## Introduction

Neurodegenerative diseases like Alzheimer’s disease (AD), Parkinson’s disease (PD), and frontotemporal dementia (FTD) pose major challenges as the global population ages (Ali et al. 2025; Basri et al. 2026; Alshehri et al. 2025; Alshahrani et al. 2025). For decades, the development of therapies has largely followed a neuron-focused approach, mainly aimed at removing protein aggregates such as amyloid-beta (Aβ) and hyperphosphorylated tau (Hayashide et al. 2026; Zhou et al. 2023; Alqahtani et al. 2025). Although newer immunotherapies targeting Aβ have shown some clinical promise, their limited effectiveness and persistent issues with dose-limiting side effects, such as amyloid-related imaging abnormalities (ARIA), suggest that neurodegeneration is not simply a proteinopathy but rather a complex, multifactorial condition involving chronic neuroinflammatory processes that contribute to disease progression (Zhang et al. 2023a; Zhao et al. 2023; Kiraly et al. 2023).

Over the last ten years, our understanding of immunity within the central nervous system (CNS) has changed dramatically. The longstanding notion that the brain is “immune-privileged” has given way to a more dynamic perspective in which the CNS and the peripheral immune system engage in active, two-way communication (Kobeissy and Salzet 2026; Castellani et al. 2023). Central to this updated framework is an integrated neuroimmune network, where resident microglia, infiltrating peripheral monocytes, adaptive T cells, and immune cells located at the meninges and choroid plexus work together to regulate responses that are either neuroprotective or neurotoxic (Müller and Di Benedetto 2025; Biswal et al. 2025; Kim and Seok 2025; Di Pietro et al. 2026). In the aging brain, a state of persistent, low-grade inflammation often arises, referred to as “inflammaging.” This condition lowers the activation threshold for microglia and perpetuates a cycle of synaptic dysfunction, neuronal injury, and progressive cognitive decline (Yang et al. 2025; Müller et al. 2025; Ishikawa et al. 2025; Malpetti et al. 2023).

Despite strong evidence connecting the state of the peripheral immune system to central nervous system (CNS) outcomes, most past anti-inflammatory treatments have focused narrowly on particular cell types or individual cytokines, which has led to poor translational success. This lack of efficacy arises from a reductionist approach that overlooks the complex, redundant nature of the neuroimmune system. As a result, targeting isolated components fails to capture the intricate interactions that influence CNS outcomes (Liu et al. 2020; Zhong et al. 2023; Mallick et al. 2025). Emerging evidence indicates that effective therapies will require the simultaneous or sequential regulation of multiple immune elements, such as T cells, B cells, and cytokines, to develop a more holistic and efficacious treatment approach (Ramírez-Valle et al. 2024; Ozog et al. 2026).

For example, recent research shows that activating the microglial NLRP3 inflammasome in tauopathies not only exacerbates local damage but also encourages T cells to infiltrate the brain tissue (Ising et al. 2019; Stancu et al. 2022).

In AD patients, clonally expanded CD8^+^ T cells have been identified in the cerebrospinal fluid (CSF), indicating that the adaptive immune system plays an active role in disease progression rather than functioning solely as a passive responder (Olst et al. 2024; Gate et al. 2020a). These insights have spurred new combination approaches that target both the innate and adaptive arms of the immune response.

This review summarizes recent advancements in therapeutically targeting integrated neuroimmune interactions in neurodegenerative diseases. The text begins by outlining the conceptual basis of the neuroimmune cell network, emphasizing the functional relationships among microglia, astrocytes, peripheral macrophages, and T cell subsets. Subsequently, it rigorously assesses innovative pharmacological strategies that utilize this integrated system, including NLRP3 inflammasome inhibitors (e.g., NT-0796), currently in Phase 1b trials for PD (Clarke et al. 2025), advanced immune checkpoint modulators that modulate T cell infiltration into the CNS (Duggan et al. 2025; Jackson et al. 2026), and groundbreaking antibodies such as ONC-841, which obstructs inhibitory SIGLEC10 receptors on microglia to improve the clearance of protein aggregates (Wang et al. 2025a).

It examines the therapeutic potential of targeting the IL-20 cytokine family, a recently discovered peripheral signaling axis that affects blood-brain barrier (BBB) integrity and microglial function (Goleij et al. 2025; Dayton et al. 2021). Ultimately, it highlights the essential requirement for dependable neuroimmune biomarkers to facilitate patient stratification, validate target engagement, and expedite clinical translation. A shift from a microglia-centric perspective toward an integrated, systems-level immunopharmacological framework is proposed, providing a timely roadmap for developing next-generation disease-modifying therapies for AD, PD, and related neurodegenerative disorders.

## From Microglia-Centric to Neuroimmune Networks

For almost twenty years, research on neuroinflammation in neurodegeneration has been primarily centered on microglia. Microglia, the brain’s intrinsic myeloid cells, are appropriately positioned at the core of disease pathogenesis: their activation states, phagocytic abilities, and inflammatory cytokine profiles are associated with Aβ accumulation, tau dissemination, and neuronal degeneration in AD, PD, and related conditions (Gao et al. 2023; Kouli et al. 2024). This emphasis produced essential insights, notably the discovery of TREM2, CD33, and other microglial risk genes associated with late-onset AD, as well as The confirmation of microglial NLRP3 inflammasome activation as a key contributor to neuroinflammatory cascades that may promote neurodegenerative processes. Nonetheless, a growing body of evidence suggests that concentrating exclusively on neuroinflammation via microglia is inadequate and potentially deceptive for therapeutic progress, as it overlooks the roles of other immune cells and signaling pathways that are vital in neurodegenerative diseases (Hammond et al. 2019; Fornari Laurindo et al. 2024).

This section consolidates the rationale for moving beyond a microglia-centric viewpoint and introduces the concept of integrated neuroimmune networks that include adaptive immune cells, border-associated macrophages (BAMs), and peripheral immune signals.

### The constraints of an exclusively microglia-centered model

Several converging lines of evidence challenge the sufficiency of the microglia-centric model. First, genetic and single-cell transcriptomic studies have revealed that disease-associated microglia (DAM) are not a homogeneous or deterministic cell state; rather, they coexist with other myeloid populations, including BAMs in the meninges, choroid plexus, and perivascular spaces, which show distinct transcriptomic signatures and functional responses in aging and neurodegeneration (Martins-Ferreira et al. 2025; Cheng and Ho 2025; Silvin et al. 2023).

It is important to recognize that microglia are not a uniform population. Single-cell transcriptomic studies have revealed considerable heterogeneity in microglial states, ranging from homeostatic surveillance phenotypes to DAM and neurodegenerative microglia (MGnD) profiles. These states exhibit context-dependent functions: in certain conditions, microglial activation promotes neuroprotection through enhanced phagocytosis and trophic factor release, while in others, it exacerbates pathology through chronic inflammatory mediator production. This functional duality emphasizes the necessity of nuanced therapeutic approaches that promote beneficial microglial functions while limiting detrimental inflammation (Keren-Shaul et al. 2017; Olah et al. 2020).

Perhaps most critically, the microglia-centric view has historically minimized the role of the adaptive immune system. Recent work has overturned this assumption: in tauopathy mouse models, microglial activation is necessary but not sufficient for T-cell recruitment, and once CD8^+^ T cells enter the parenchyma, they directly contribute to neuronal damage independent of microglial state (Chen et al. 2023). Similarly, single-cell analyses of CSF from AD patients have identified clonally expanded CD8^+^ T cells with an antigen-experienced, cytotoxic phenotype, indicating an active adaptive immune response within the CNS (Zhao et al. 2026a; Peng et al. 2026a). These findings collectively suggest that microglia represent a significant component within a broader neuroimmune network.

### An emerging model: the connection between neural and immune networks

An innovative conceptual framework, the integrated neuroimmune network, is developing in reaction to these limitations. This paradigm asserts that neurodegeneration results from dysregulated interactions among various immune components, including CNS-resident microglia and BAMs, infiltrating peripheral T cells and monocytes, along with systemic immune signals conveyed through the gut-brain axis, meningeal lymphatics, and the choroid plexus (Müller et al. 2025; Peng et al. 2026a; Carloni and Rescigno 2022).

Available evidence suggests that no single cell type solely drives this network; rather, feedback loops and feed-forward amplification cycles propagate neuroinflammation. The release of IL-1β by microglia can compromise the integrity of the BBB, enabling T-cell infiltration; subsequently, IFN-γ produced by T-cells further activates microglia and amplifies antigen presentation, thereby perpetuating the cycle (Wang et al. 2014; Fetsko et al. 2024).

This network perspective has significant therapeutic ramifications. Focusing on a single node, like depleting microglia or blocking a certain cytokine, may not be enough if other nodes are still active or are compensating by increasing their activity. On the other hand, interventions that restore communication balance within the network (for example, improving Treg function while changing microglial priming) may have longer-lasting effects. Recent data corroborate this perspective: in a Phase 1b trial, the CNS-penetrant NLRP3 inhibitor NT-0796 not only diminished microglial IL-1β but also normalized cerebrospinal fluid markers of T-cell activation and axonal injury in patients with PD, indicating that targeting a central myeloid node can affect the broader immune network (Clarke et al. 2025).

### Essential non-microglial cell types and their intercellular signaling routes

A comprehensive understanding of the integrated neuroimmune network requires appreciating the roles of at least three additional players, which include BAMs that are transcriptionally distinct from microglia and exhibit higher turnover rates, greater dependence on peripheral monocytes, and unique responses to aging and disease (Da Mesquita and Rua 2024; Wang et al. 2024). In AD mouse models, BAMs have been shown to internalize Aβ from the perivascular space, and their dysfunction correlates with cerebral amyloid angiopathy (Zhan et al. 2025a; Uekawa et al. 2023). Because BAMs are more accessible to systemically administered drugs than parenchymal microglia, they represent an attractive therapeutic target (Zheng et al. 2025).

Adaptive T lymphocytes such as CD4^+^ T cells (particularly Th1 and Th17 subsets) and CD8+ T cells infiltrate the CNS in AD, PD, and ALS (Sacharczuk et al. 2024; Kim et al. 2024; Zhang et al. 2023b; Terrabuio et al. 2023). Importantly, antigen-specific T-cell responses against Aβ, tau, and α-synuclein have been detected in both mice and humans (Chiu et al. 2025; Dhanwani et al. 2020). Regulatory T cells (Tregs), in contrast, exert neuroprotective effects by suppressing microglial activation and limiting parenchymal T-cell infiltration (Liu et al. 2023; Prasad et al. 2023). Preclinical expansion of Tregs via low-dose IL-2 or CCR4 antagonists has shown benefit in AD and PD models, and early-phase clinical trials are underway (Faridar et al. 2023; Markovic et al. 2022).

The peripheral-to-CNS signaling axis, like systemic inflammation (e.g., from infection, surgery, or metabolic syndrome), can exacerbate neurodegeneration via humoral, cellular, and neural pathways (Zhang et al. 2023a; Paouri and Georgopoulos 2019).

The IL-20 cytokine family, for instance, has recently emerged as a key mediator of peripheral-to-CNS crosstalk: IL-19, IL-20, and IL-24 are upregulated in the brains of AD and PD patients in exploratory studies, modulate microglial activation and BBB integrity in preclinical models, and are druggable with existing monoclonal antibodies. However, the clinical utility of these molecules as biomarkers remains to be established in larger, well-controlled longitudinal cohorts (Goleij et al. 2025; Chen et al. 2018; Tylutka et al. 2024).

**Fig 1:**
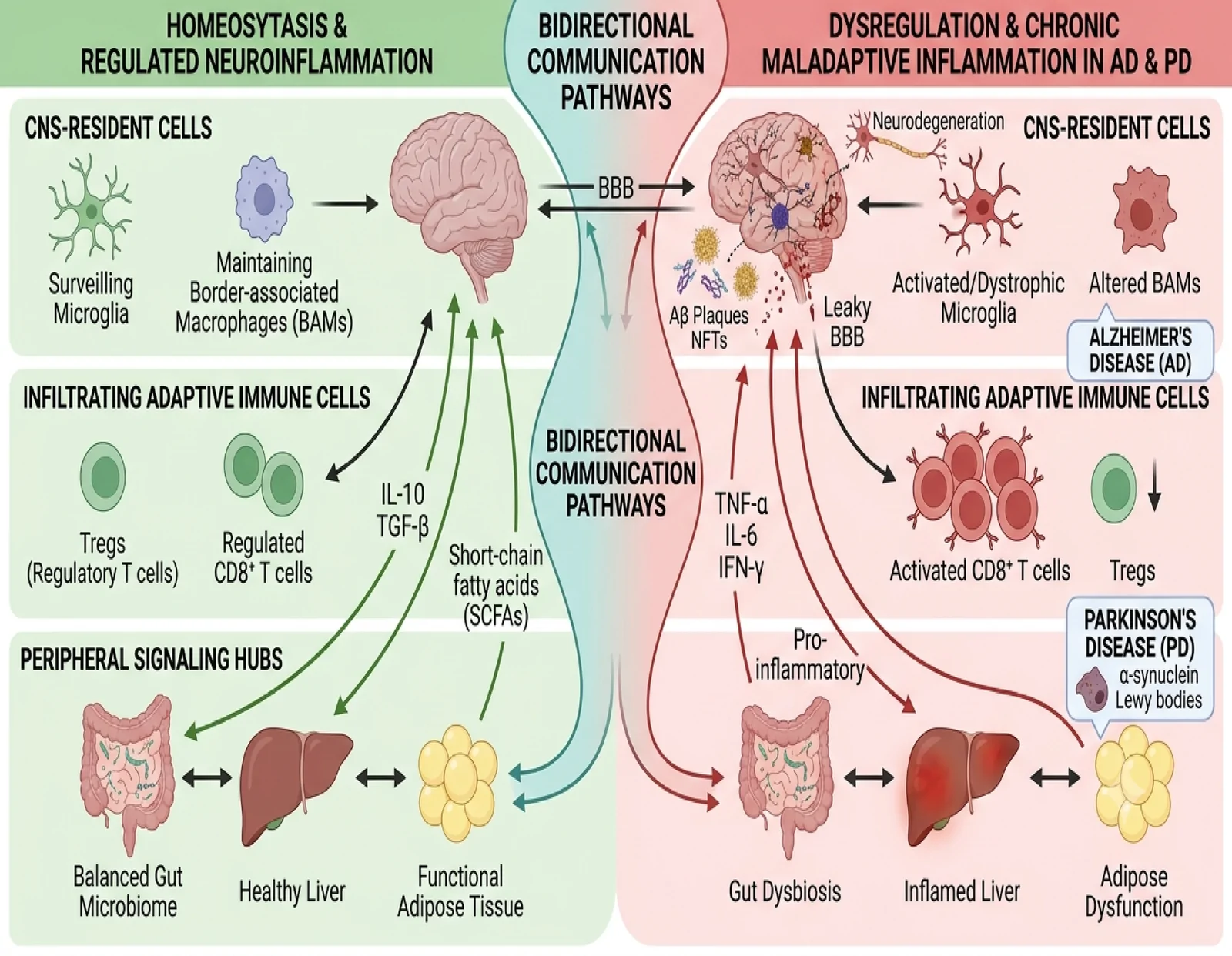
Dual-state model of neuroinflammation in neurodegenerative diseases. This schematic illustrates the balance between homeostatic regulation and chronic dysregulation within the CNS and peripheral systems. In the homeostatic state **(left)**, CNS-resident cells (surveilling microglia and border-associated macrophages) maintain tissue integrity alongside infiltrating regulatory T cells (Tregs) and regulated CD8+ T cells. This equilibrium is supported by peripheral feedback, including a balanced gut microbiome, healthy liver function, and functional adipose tissue, operating through bidirectional communication pathways at the BBB and soluble mediators. In contrast, the dysregulated state **(right)** is characterized by maladaptive inflammation, driven by activated/dystrophic microglia and pro-inflammatory CD8+ T cells. This dysregulated state is associated with pathological hallmarks such as amyloid-β plaques and NFTs in AD and α-synuclein Lewy bodies in PD. Peripheral co-morbidities, including gut dysbiosis, hepatic inflammation, and adipose dysfunction, contribute to a pro-inflammatory milieu, driving neurodegeneration and disease progression

Similarly, the gut microbiome produces metabolites (e.g., short-chain fatty acids) that influence microglial maturation and T-cell polarization, linking peripheral ecology to CNS immune tone (Loh et al. 2024; Warren et al. 2024; Colombo et al. 2021) (Fig. 1). Furthermore, microglial heterogeneity and context-dependent responses present additional complexity. The same therapeutic intervention may yield divergent outcomes depending on the predominant microglial state within an individual patient, emphasizing the necessity for detailed endotyping prior to therapy selection.

### Immunopharmacological ramifications

Moving beyond the microglia-centric view directly informs drug discovery and clinical trial design. First, biomarker strategies must diversify; relying solely on microglial markers (e.g., TSPO-PET) risks missing adaptive immune or BAM contributions (Noh et al. 2025).

Instead, multiplex panels measuring CSF cytokines (e.g., IL-6, IFN-γ, IL-17), T-cell receptor clonality, and BAM-derived proteins (e.g., CD163) are needed (Sepiashvili et al. 2021). Second, combination therapies may be required: an NLRP3 inhibitor might be paired with a Treg enhancer or a peripheral anti-inflammatory agent (e.g., an IL-20 family blocker) to simultaneously target multiple network nodes. Third, patient stratification based on neuroimmune endotypes, e.g., microglia-dominant vs. T-cell-dominant inflammation, could improve trial outcomes, a strategy already being explored in PD and multiple sclerosis (MS) (Müller and Di Benedetto 2025; Cabral et al. 2025; Isik et al. 2023; Schetters et al. 2018; Nan et al. 2024).

**Fig 2:**
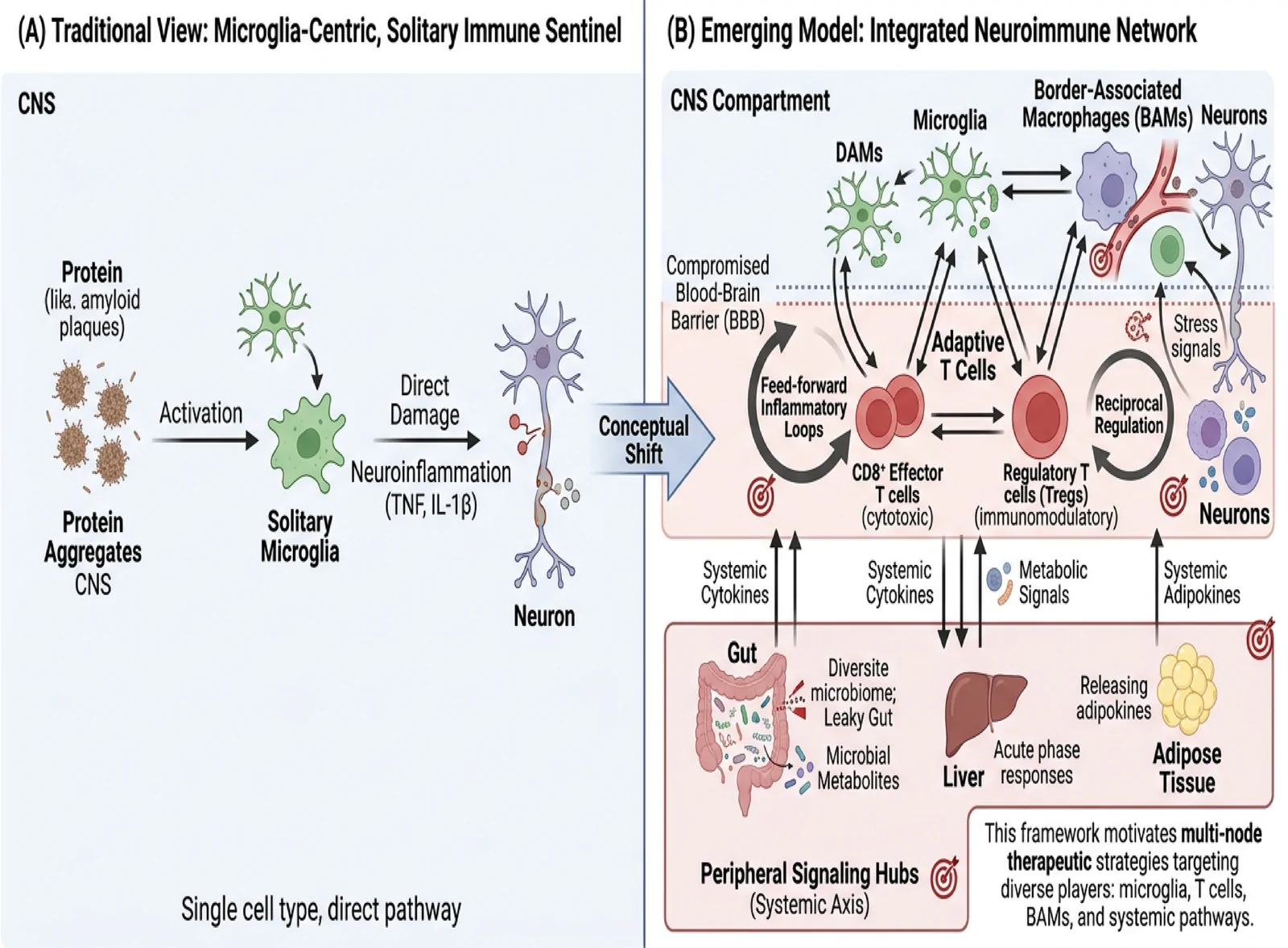
Transitioning from a Microglia-Focused to a Comprehensive Neuroimmune Network Framework of Neurodegeneration. The conventional perspective regards microglia as the principal and exclusive immune guardian, directly reacting to protein aggregates (amyloid-β, tau, and α-synuclein) and resulting in neuronal harm (denoted by the red lightning bolt). This model neglects the roles of adaptive immunity and peripheral signaling. The nascent integrated network framework includes CNS-resident microglia, border-associated macrophages, infiltrating adaptive T cells (CD8⁺ effectors and Tregs), and peripheral signaling hubs (gut-liver-adipose axes). Bidirectional arrows signify mutual regulation and feed-forward inflammatory circuits. Cerulean arrows denote protective interactions; crimson arrows signify pathogenic amplification. This framework encourages multi-node therapeutic approaches, such as NLRP3 inhibition, Treg augmentation, and peripheral immunomodulation

In summary, the integrated neuroimmune network framework does not diminish the importance of microglia but rather places them in their proper biological context. The remainder of this review will explore specific therapeutic strategies that arise from this paradigm, including targeting T-cell subsets, modulating BAM function, and intercepting peripheral-to-CNS signals, all while acknowledging that successful immunopharmacology in neurodegeneration will require network-aware, multi-node interventions (Table 1 and Fig. 2).

**Table 1:** Essential elements of the integrated neuroimmune network. Location, function, dysfunction, and therapeutic approaches aimed at microglia, border-associated macrophages (BAMs), T cell subsets, and peripheral signaling hubs in AD and PD

Component	Location	Primary function	Dysfunction in neurodegeneration	Therapeutic strategies	Ref
Microglia	CNS parenchyma	Synaptic pruning, debris clearance, immune surveillance	Chronic NLRP3 activation, dystrophic morphology, impaired phagocytosis	NLRP3 inhibitors (NT-0796)	Clarke et al. 2025; Harrison et al. 2023)
Border-associated macrophages (BAMs)	Meninges, choroid plexus, perivascular spaces	Antigen presentation, BBB maintenance, CSF surveillance	Dysfunction correlates with cerebral amyloid angiopathy	Systemically accessible targets; repurposed biologics	Sun and Jiang 2024)
CD8^+^ T cells	Infiltrating parenchyma and CSF	Cytotoxic elimination of infected/malignant cells	Clonal expansion, granzyme B/perforin release, neuronal damage	Treg enhancement (low-dose IL-2),	Zhang et al. 2023b; Harris et al. 2023; Rosenzwajg et al. 2015; Wang et al. 2022; Zhao et al. 2025; Faridar et al. 2025)
CD4^+^ T cells (Th1/Th17)	Infiltrating parenchyma	Helper functions, cytokine secretion (IFN-γ, IL-17)	Pro-inflammatory polarization, microglial activation	Antigen-specific tolerance induction	Yu et al. 2024; Faas et al. 2025a; Yoon et al. 2025)
Regulatory T cells (Tregs)	Infiltrating parenchyma	Suppression of effector T cells, resolution of inflammation	Reduced numbers/function, inadequate suppression	Low-dose IL-2, CCR4 antagonists	Dowling et al. 2018; Ketcham et al. 2018; Bogacka et al. 2022)
Peripheral signaling hubs	Blood, gut, liver, adipose tissue	Systemic immune-metabolic communication	Elevated IL-19/IL-20, SCFA dysregulation, leptin/adiponectin imbalance	Anti-IL-20 antibodies, FGF21 analogs	Goleij et al. 2025; Giannoni et al. 2020; Park et al. 2020; Strauss et al. 2025; Yang et al. 2021; Kang et al. 2020; Fawzy and Fathy 2026)

Having established the integrated neuroimmune network framework and its immunopharmacological implications, now turn to the most transformative recent insight in this paradigm: the recognition that adaptive immunity, particularly clonally expanded T cells, actively drives neurodegeneration rather than merely responding to it. This mechanistic understanding directly informs novel therapeutic strategies that modulate T cell trafficking, function, and antigen specificity, approaches that are now entering early-phase clinical trials.

## Adaptive immunity in neurodegeneration

However, as outlined in Sect. "From Microglia-Centric to Neuroimmune Networks", this microglia-centric view has gradually been replaced by a more integrated perspective, in which peripheral immune cells, especially T lymphocytes, actively contribute to and sustain neurodegeneration (Ana 2024). This section consolidates the accumulating evidence suggesting that adaptive immunity is not a delayed secondary phenomenon but an early, mechanistic catalyst of diseases such as AD, PD, and tauopathies. The discourse emphasizes three principal advancements: the identification of clonally expanded T cells in the CNS, the antigen-specific characteristics of these immune responses, and the functional implications of the interaction between T cells and microglia.

### Expanded clones of CD8^+^ T cells found in the AD brain

The most transformative insight in recent years has come from single-cell transcriptomic and T cell receptor (TCR) sequencing of postmortem AD brain tissue and CSF (Wang et al. 2021; Ramos-Vicente et al. 2025). Multiple independent studies have now demonstrated that the AD brain harbors clonally expanded CD8^+^ T cells, a hallmark of antigen-driven adaptive immune responses. These cells are not merely passive infiltrators; they express cytotoxic effector molecules, including granzyme B, perforin, and IFN-γ, and are enriched in close proximity to amyloid plaques and tau-laden neurons (Gate et al. 2020b; Panwar et al. 2024; Ohyagi et al. 2025).

Critically, longitudinal analyses of CSF from living AD patients have revealed that the magnitude of CD8^+^ T cell clonal expansion correlates with the rate of cognitive decline and with CSF markers of axonal injury (e.g., neurofilament light chain). These findings elevate T cells from bystanders to active participants in disease progression (Gate et al. 2020b; Peng et al. 2026b). Moreover, the TCR repertoires of these cells show convergent public clonotypes across different patients, suggesting recognition of a shared set of CNS-derived antigens (Xue et al. 2025; Planas et al. 2018). While the precise nature of these antigens remains under active investigation, candidates include post-translationally modified tau, amyloid-β-derived peptides, and neoepitopes generated by oxidative damage, all of which accumulate with age and disease (Song et al. 2024; Karapetyan et al. 2022).

### T Cell responses targeting specific antigens in tauopathy and PD

The function of adaptive immunity extends beyond AD. In tauopathy models, including P301S tau transgenic mice, CD8⁺ T cells invade the brain in a manner that is dependent on the disease stage (Holtzman 2024). The reduction of CD8⁺ T cells in these subjects mitigates neurodegeneration, decreases tau hyperphosphorylation, and maintains synaptic integrity (Terrabuio et al. 2025).

Microglia activated by pathological tau present antigens through MHC class I, directly engaging and activating CD8⁺ T cells (Chen et al. 2023; Askin and Wegmann 2023). The microglia–T cell axis creates a positive feedback loop: activated microglia recruit and proliferate cytotoxic T cells, which subsequently release pro-inflammatory cytokines that further activate microglia (Groh et al. 2025). The evidence in PD is equally compelling. α-Synuclein, the protein that accumulates in Lewy bodies, has been shown to act as a neoantigen. Peripheral T cells from a specific subset of PD patients exhibit unique reactivity to α-synuclein-derived epitopes, particularly those containing phosphorylated or nitrated residues, modifications commonly found in affected brains (Sulzer et al. 2017; Garretti et al. 2019).

Furthermore, postmortem brains of individuals with PD display an accumulation of CD8⁺ T cells surrounding dopaminergic neurons in the substantia nigra (Ma et al. 2025; Galiano-Landeira et al. 2020). Single-cell analyses confirm that these cells possess cytotoxic mechanisms, and their abundance inversely correlates with the number of remaining tyrosine hydroxylase-positive neurons (Wang et al. 2021; Hu et al. 2022). Together, these data indicate that adaptive immune responses to disease-specific protein aggregates are a common feature across multiple neurodegenerative conditions.

### Pathways governing T cell infiltration, prolonged stay, and functional activity in the CNS

For T cells to contribute to neurodegeneration, they must first access the CNS parenchyma (Chen et al. 2023). Under physiological conditions, the BBB, and the blood**-**CSF barrier restrict lymphocyte entry (Ayub et al. 2021). However, in the aging and degenerating brain, the BBB becomes progressively compromised (Knox et al. 2022). Emerging evidence indicates that even subtle BBB dysfunction, detectable in mild cognitive impairment, permits the extravasation of activated T cells (Barisano et al. 2022; Zeng et al. 2024).

Once inside the perivascular spaces or CSF, T cells encounter antigen-presenting cells, including perivascular macrophages and infiltrating dendritic cells, as well as microglia themselves. Antigen recognition initiates T cell activation, proliferation, and retention (Qin et al. 2021; Goddery et al. 2021). Cytotoxic CD8^+^ T cells subsequently interact with neurons or glia presenting cognate antigens through MHC class I. Neurons are not passive targets; during inflammatory stress, they increase MHC class I expression, making them vulnerable to T cell-mediated destruction (Chevalier et al. 2011; Clarkson et al. 2023). This mechanism may elucidate the localized neuronal degeneration typical of numerous neurodegenerative disorders.

### Clinical potential of modulating adaptive immune responses

The recognition that adaptive immunity actively drives neurodegeneration has opened entirely new therapeutic avenues, with several strategies now entering preclinical and early clinical testing (Ahn and Shin 2026). Modulation of T cell trafficking via blockade of chemokine receptors (e.g., CCR2, CXCR3) or integrins (e.g., VLA-4) can limit T cell entry into the CNS; although these approaches have shown promise in MS, their application in AD and PD requires careful timing to avoid impairing immune surveillance. Another important strategy is to improve Treg function (Parween et al. 2025).

For example, giving low doses of interleukin-2 (IL-2) to increase the number of Tregs reduces neuroinflammation, lowers T cell infiltration, and improves cognitive or motor outcomes in models of AD and PD (Markovic et al. 2022; Faridar et al. 2025). Phase I trials of low-dose IL-2 in AD are currently underway (Faridar et al. 2025).

**Fig 3:**
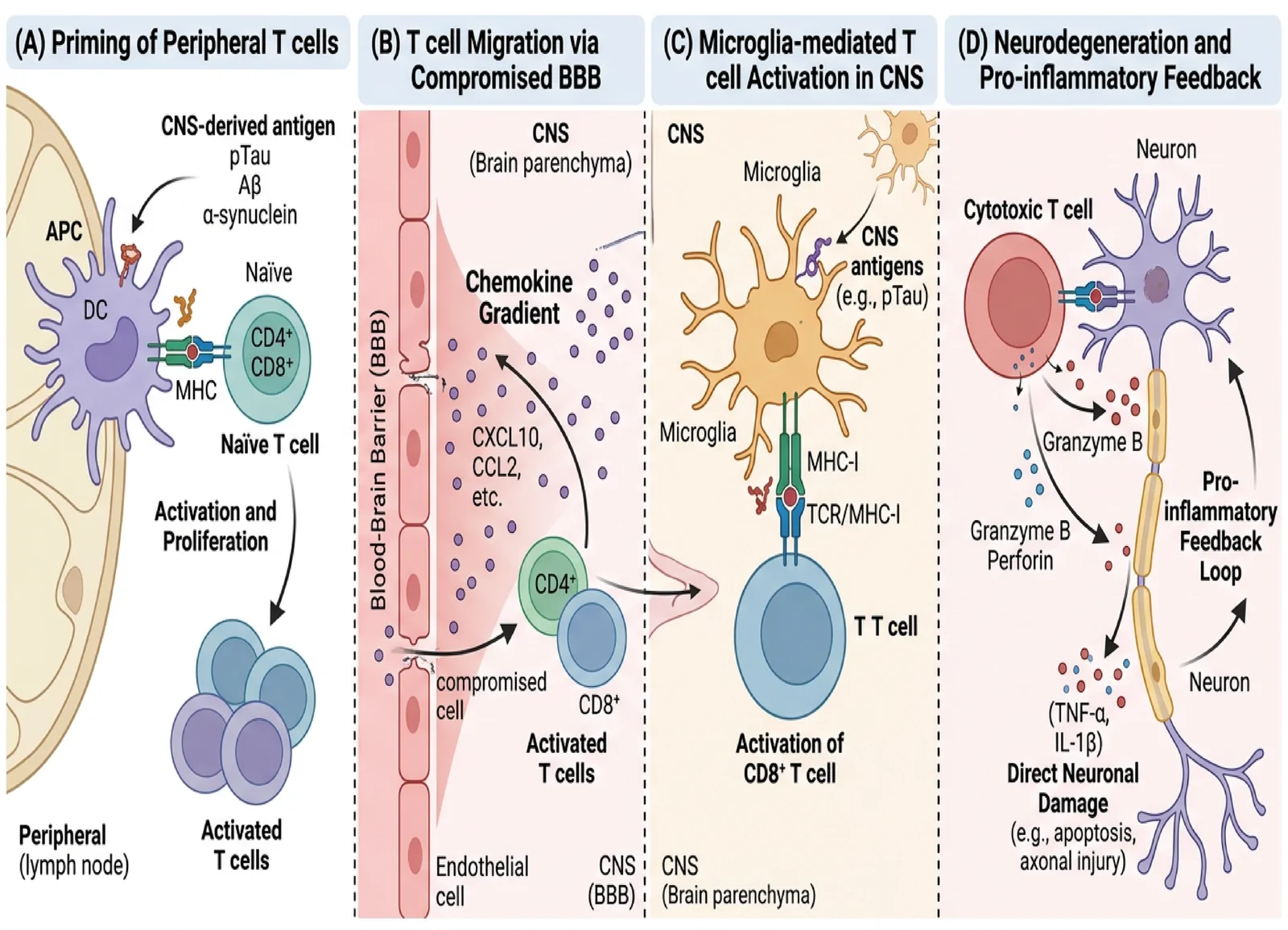
Mechanisms of T Cell-Mediated Neurodegeneration. Schematic illustrating the sequence of T cell-mediated neurotoxicity in neurodegenerative diseases. (A) Peripheral T cells are primed against CNS-derived antigens (pTau, Aβ, α-synuclein) in secondary lymphoid organs. (B) Activated T cells cross the compromised BBB via chemokine gradients (CXCL10, CCL2) and integrin-mediated adhesion (VLA-4/VCAM-1). (C) Within the CNS, microglia present antigens via MHC class I, directly engaging and activating CD8⁺ T cells (indicated by TCR-MHC interaction). (D) Cytotoxic T cells release granzyme B and perforin, directly damaging neurons and creating a pro-inflammatory feedback loop. Red arrows indicate pro-inflammatory pathways; green arrows indicate regulatory pathways (Treg-mediated suppression)

More precise methodologies encompass antigen-specific tolerance induction, employing mucosal administration or engineered tolerogenic vaccines to eliminate pathogenic T cell clones targeting disease-relevant antigens like α-synuclein or tau peptides while avoiding widespread immunosuppression (Tan et al. 2024; Zhan et al. 2025b). Checkpoint modulation necessitates prudence: while immune checkpoint inhibitors can provoke significant neuroinflammatory side effects, the activation of checkpoint pathways (e.g., PD-1/PD-L1) may attenuate pathogenic T cell responses in neurodegenerative conditions (Moadab et al. 2025). Preclinical data indicate that PD-L1-Fc fusion proteins diminish T cell infiltration and alleviate tau pathology, underscoring a promising avenue for future therapeutic advancement (Harris et al. 2025) (Fig. 3).

While the preceding section focused on adaptive immune cells that infiltrate the CNS, an equally critical component of the neuroimmune network operates upstream of CNS entry: soluble signaling molecules derived from peripheral tissues. These peripheral hubs, including the IL-20 cytokine family, gut microbiome metabolites, and hepatokine networks, offer accessible therapeutic targets that can modulate neuroinflammation before it reaches the brain parenchyma. The following section examines these peripheral-to-central signaling pathways as promising immunopharmacological intervention points.

## Peripheral-central signaling hubs as therapeutic targets

While adaptive immune cells infiltrating the CNS facilitate direct neuroimmune interactions, an equally crucial component of neuroinflammation involves soluble signaling molecules derived from the periphery that functionally modify the CNS. These signaling hubs, comprising cytokine families, gut-derived metabolites, and hepatokine networks, operate as integrated communication systems that can either preserve or disrupt brain homeostasis (Müller and Benedetto 2025). Their accessibility to pharmacological treatment makes them particularly attractive therapeutic targets for neurodegenerative disorders.

### The emerging role of the IL-20 family in the neuroimmune interface

Among the most promising but underappreciated peripheral signaling systems is the IL-20 cytokine subfamily, which includes IL-19, IL-20, IL-22, IL-24, and IL-26. Historically studied for their roles in epithelial barrier function and psoriasis (Wolk et al. 2009; Rutz et al. 2014), recent evidence has positioned this family as a key regulator of neuroinflammation. Unlike classical pro-inflammatory cytokines such as IL-1β and TNF-α, IL-20 family members exhibit complex, context-dependent signaling through heterodimeric receptors (IL-20R1/IL-20R2 and IL-22R1/IL-20R2) that are expressed not only on peripheral immune cells but also on brain endothelial cells, astrocytes, and microglia (Goleij et al. 2025; Dayton et al. 2021).

In animal models of tauopathy, neutralization of IL-20 signaling reduces microglial NLRP3 inflammasome activation and ameliorates synaptic loss independent of amyloid pathology (Ising et al. 2019; Zhang et al. 2024a). These findings suggest that targeting the IL-20 axis with clinically available monoclonal antibodies (e.g., anti-IL-20 antibodies in phase II trials for psoriasis) could be repurposed for neurodegeneration, a classic immunopharmacology opportunity (Dayton et al. 2021; Lundblad et al. 2015).

### The gut-brain link: microbial metabolites as key master switches

No discussion of peripheral-to-central signaling is complete without addressing the gut-brain axis, arguably the most integrated peripheral signaling hub. The gut microbiome produces hundreds of metabolites, short-chain fatty acids (SCFAs) (SCFAs; acetate, propionate, and butyrate), tryptophan derivatives (indoles and kynurenine), and secondary bile acids that reach the CNS via the systemic circulation and modulation neuroinflammation through distinct molecular mechanisms (Cryan et al. 2019; Mu and Wang 2025).

SCFAs have emerged as dual-purpose agents. In contrast, tryptophan-derived indoles, especially indole-3-propionic acid (IPA), exhibit neuroprotective properties by inhibiting astrocytic NF-κB activation and maintaining blood-brain barrier integrity (Owe-Larsson et al. 2025). The therapeutic targeting of this hub is currently progressing in clinical trials. Orally administered Clostridium butyricum, a butyrate-producing bacterium, diminished neuroinflammation and enhanced motor outcomes in a preclinical model of PD (Sun et al. 2021), while fecal microbiota transplantation from healthy donors is being studied for early AD (Ren et al. 2025; Upadhyay et al. 2025).

These microbiome-modulating strategies signify a change in basic assumptions: instead of directly inhibiting a cytokine or immune receptor, they reconfigure the peripheral signaling milieu upstream of CNS pathology.

### Hepatokines and neurodegeneration: insights from the liver-brain axis

The liver, serving as the body’s metabolic monitor, secretes signaling proteins known as hepatokines that communicate nutritional and inflammatory conditions to distant organs, including the brain. Two hepatokines have garnered considerable attention in the context of neurodegeneration: fibroblast growth factor 21 (FGF21) and alpha-1-antitrypsin (AAT) (Yang et al. 2024; Chen et al. 2019; Zhou et al. 2018). Serum FGF21 levels are significantly increased in prodromal AD but decrease in moderate-to-severe stages, indicating a compensatory mechanism that proves inadequate (Dallaire‐Théroux et al. 2026). Recombinant FGF21 analogs (e.g., PF-05231023) have been evaluated in metabolic disorders and may be repurposed for the initial stages of neurodegeneration (Zhao et al. 2024). AAT, an acute-phase protein synthesized by hepatocytes, is primarily recognized as a serine protease inhibitor that safeguards lung tissue in emphysema (Wang et al. 2025b). AAT also exhibits significant anti-inflammatory and immunomodulatory effects that are independent of protease inhibition (Jonigk et al. 2013).

In preclinical models of AD, intravenous AAT therapy diminishes microglial MHC-II expression, decreases IL-1β and IL-6 production, and fosters a phagocytic microglial phenotype that eliminates Aβ (Hassan et al. 2025; Sun et al. 2024). Therefore, may be repurposed for the initial stages of neurodegeneration.

### Avenues for intervention and unresolved problems

The convergence of these peripheral signaling hubs presents a rich pipeline for immunopharmacology. Several therapeutic strategies are immediately translatable: repurposing of existing biologics (anti-IL-20 antibodies), microbiome-targeted interventions (defined bacterial consortia, prebiotics), and hepatokine analogs (recombinant FGF21, plasma-derived AAT). What unites these approaches is their targeting of pathways upstream of CNS inflammation, peripheral signals that can be modified without crossing the BBB in some cases, or that cross the BBB through endogenous transport mechanisms.

However, significant deficiencies persist. The contribution of each peripheral hub differs according to disease stage and individual genetic predisposition, requiring biomarker-based patient stratification. It is important to emphasize that many candidate biomarkers discussed in this review, including IL-20 family cytokines, remain at an exploratory stage. Although they demonstrate promise in pilot studies, their clinical utility requires rigorous prospective validation in large, diverse, well-characterized patient cohorts with longitudinal follow-up. Standardized assays and predefined cutoffs are needed before these markers can be recommended for clinical decision-making. Second, peripheral signaling exhibits significant redundancy; inhibition of one cytokine may be offset by another. Third, longitudinal studies are essential to ascertain whether peripheral abnormalities are causal or merely correlative. Fourth, the safety of chronic peripheral immunomodulation in elderly populations, who are already immunosenescent, necessitates meticulous assessment.

The therapeutic potential of targeting peripheral signaling hubs, while conceptually compelling, remains largely at the preclinical or exploratory stage. The ultimate validation of any neuroimmune therapeutic framework requires demonstration of clinical efficacy in human trials. Now examine the most advanced investigational agents that have progressed to clinical development, focusing on those designed to penetrate the CNS and directly modulate neuroinflammatory pathways, thereby providing a practical test of the principles discussed thus far.

Despite these challenges, the peripheral-to-central signaling framework reframes neurodegeneration as a systemic disorder rather than an isolated brain disease (Table 2).

**Table 2:** Peripheral-to-central signaling hubs in neurodegeneration: mechanisms, neuroinflammatory effects, and therapeutic opportunities

Signaling hub	Key mediators	Mechanism of CNS action	Effect on neuroinflammation	Therapeutic strategy	Clinical status	Refs
IL-20 cytokine family	IL-19, IL-20, IL-24	Cross BBB; induce astrocytic CCL2/CXCL10; modulate microglial NLRP3	Pro-inflammatory (elevated in AD/PD in exploratory studies)	Anti-IL-20 mAbs (repurposed from psoriasis)	Preclinical; exploratory biomarker studies ongoing	Goleij et al. 2025; Gottlieb et al. 2015; Hsu and Chang 2014)
Gut-brain axis	SCFAs (butyrate, propionate), indoles (IPA)	SCFAs: histone acetylation of pro-inflammatory genes; IPA: inhibit NF-κB	Dual: protective (Treg) vs. harmful (microglial activation)	FMT, *Clostridium butyricum*, prebiotics	Phase II (PD)	Colombo et al. 2021; Wachamo and Gaultier 2025; Silva et al. 2020; Li et al. 2025; Hegelmaier et al. 2025; Sun et al. 2020)
Liver-brain axis	FGF21, AAT	FGF21: crosses BBB, inhibits microglial IFN-γ; AAT: reduces MHC-II, IL-1β/IL-6	Protective (compensatory, then inadequate)	Recombinant FGF21 analogs; plasma-derived AAT	Preclinical; AD	Zhao et al. 2026b; Wang et al. 2025c; Cummings 2024)
Adipose-brain axis	Leptin, adiponectin	Leptin: pro-inflammatory (microglial activation, Th17); Adiponectin: anti-inflammatory (AMPK-mediated phagocytosis)	Leptin harmful; adiponectin protective (limited BBB penetration)	Leptin receptor antagonists; AdipoR agonists (AdipoRon)	Preclinical	Altarejos et al. 2023; Zabeau et al. 2019; Liu and Li 2025)

## Clinical trials of first-in-class neuroimmune agents

The translation of fundamental neuroimmune insights in clinical practice signifies the pinnacle of validation for the therapeutic paradigm. This section emphasizes agents that have progressed to human trials, particularly those intended to infiltrate the CNS and directly influence neuroinflammatory pathways, following the discussion in Section "Peripheral-central signaling hubs as therapeutic targets" regarding the potential of peripheral-to-central signaling hubs like the IL-20 cytokine family.

The recent completion of first-in-human and proof-of-concept studies for various CNS-penetrant, anti-neuroinflammatory compounds represents a significant advancement in the field, transforming a previously theoretical notion into a tangible clinical reality.

### The NLRP3 inflammasome: moving from a preclinical focus to a clinical standard

The NLRP3 (NACHT, LRR, and PYD domains-containing protein 3) inflammasome is one of the most vigorously investigated components in central neuroinflammation (Xu et al. 2025; El-Sayed et al. 2023; Fawzy et al. 2025, 2026). This multiprotein oligomer, activated in microglia by Aβ, alpha-synuclein, tau aggregates, and other DAMPs, catalyzes the maturation and secretion of IL-1β and IL-18, thus perpetuating a robust feed-forward inflammatory loop (Zhang et al. 2024a; Auger et al. 2025; Pike et al. 2021). Preclinical investigations utilizing NLRP3 knockout mice or small-molecule inhibitors (e.g., MCC950, glyburide) exhibited significant neuroprotection in models of AD, PD, MS, and traumatic brain injury (Corcoran et al. 2021; Xu et al. 2018). Nevertheless, the translation of these findings encountered significant obstacles: first-generation inhibitors demonstrated inadequate CNS bioavailability, off-target toxicity, or metabolic instability, with MCC950 being discontinued due to hepatotoxicity signals observed in Phase I trials (Shi et al. 2023; Chen et al. 2024).

The landscape evolved with the development of next-generation, CNS-optimized NLRP3 inhibitors, although all remain investigational agents. NT-0796 (NodThera, Inc.) represents a paradigm shift in design philosophy: this oral, peripherally restricted prodrug was engineered for rapid conversion to its active moiety within the CNS, achieving brain-to-plasma ratios exceeding 2:1 in preclinical species, a feat rarely accomplished for small-molecule inflammasome inhibitors (Harrison et al. 2023).

In a recently completed Phase 1b/2a clinical trial (NCT05453968) enrolling patients with mild-to-moderate PD, the investigational agent NT-0796 met its primary safety endpoint with a favorable tolerability profile over 28 days of dosing (Clarke et al. 2025).

Notably, exploratory biomarker analyses indicated a substantial decrease in CSF IL-1β and IL-18 (approximately 45-60% from baseline, *p* < 0.01), thereby suggesting target engagement within the CNS; normalization of CSF caspase-1 activity, a proximal indicator of inflammasome function, to levels comparable to those of age-matched healthy controls; and a reduction in CSF neurofilament light chain (NfL), a marker of axonal injury, by an average of 22% (p = 0.03), implying downstream neuroprotective effects (Clarke et al. 2025).

It is critical to emphasize the following caveats regarding these findings: (1) the Phase 1b trial was primarily designed to assess safety and tolerability, not clinical efficacy; (2) the biomarker analyses were exploratory and not pre-specified as primary endpoints; (3) the sample size was limited (n<100), and results require confirmation in larger, adequately powered studies; (4) the 28-day dosing period is insufficient to assess long-term safety or sustained disease modification; and (5) NT-0796 remains an investigational compound with no regulatory approval for PD or any other neurodegenerative indication. Furthermore, positive preclinical findings with NLRP3 inhibition have not always translated to clinical benefit, underscoring the need for confirmatory studies. A follow-up Phase 2 trial in early-stage PD is currently enrolling, with secondary endpoints including motor progression and dopaminergic imaging. Results from this larger, longer-duration study will be necessary to determine whether the promising biomarker signals observed in the Phase 1b trial translate into meaningful clinical benefits.

### Adjusting immune checkpoints in microglia: unleashing phagocytic activity

A novel therapeutic approach draws on the efficacy of immune checkpoint inhibitors for cancer treatment (Zhang et al. 2024b). Under physiological conditions, microglia express “don’t-eat-me” signals (e.g., CD47 and CD200) and their cognate receptors (e.g., SIRPα and CD200R) that limit phagocytosis of viable neurons and synapses (Ju et al. 2025).

In neurodegeneration, however, this brake system becomes pathologically engaged, preventing microglia from clearing protein aggregates (Fu et al. 2025; Muzio et al. 2021). Therapeutic blocking of these checkpoints represents an inverted strategy: rather than suppressing inflammation, the goal is to restore microglial competence for aggregate clearance (Shao et al. 2025). ONC-841 (OncoC4, formerly OncoImmune) is a first-in-class humanized monoclonal antibody targeting SIGLEC10 (sialic acid-binding Ig-like lectin 10), an inhibitory receptor expressed on microglia. Genetic research has revealed uncommon loss-of-function variants in SIGLEC10 that confer protection against late-onset AD, indicating that natural variations in this pathway influence disease susceptibility (Wang et al. 2025d).

Two AD mouse models (ARTE and JNPL3) were crossed with human SIGLEC transgenic mice to generate SIGLEC10-expressing AD models. To assess whether ONC‐841 can cross the blood–brain barrier, the antibody was administered intravenously, and occupancy of microglial SIGLEC10 was measured using flow cytometry and immunohistochemistry. To avoid xenoreactivity, a mouse IgG version of ONC‐841 (31F11) was generated and used for evaluating therapeutic effects. Brain accumulation of pTau (AT8) and amyloid plaques (6E10) was assessed via immunohistochemistry. Plasma levels of pTau181 and total tau were measured as biomarkers. The mechanism of action was explored using single-nucleus RNA sequencing and in vitro phagocytosis assays (Wang et al. 2025a). The preclinical data revealed that twenty-week-old ARTE10/Sg10 mice were administered intravenous injections of either 31F11 or vehicle control over a duration of 6.5 weeks. In comparison to controls, brain sections from treated mice exhibited a significant decrease in amyloid‐β plaque accumulation. In JNPL3/Sg10 mice, treatment with 31F11 resulted in a notable reduction in both plasma p-tau181 and total tau concentrations.

In vitro phagocytosis assays demonstrated that ONC‐841 enhanced the uptake of both Aβ and tau aggregates by human iPSC-derived microglia and primary mouse microglia. Morphological analysis confirmed these findings, demonstrating that 31F11 corrected microglial abnormalities linked to AD. Furthermore, single-nucleus RNA sequencing revealed that anti-SIGLEC10 treatment restored the phagocytic and migratory functions of microglia. These findings collectively indicate that ONC‑841 protects against AD pathology in mice by rejuvenating microglial function. ONC-841 inhibits SIGLEC10 interaction with its ligand, CD24, thereby eliminating an inhibitory signal and enhancing microglial phagocytosis (Wang et al. 2025a).

These studies established the scientific basis for the subsequent clinical advancement of ONC‐841 as a prospective microglia-targeting immunotherapeutic agent for AD. The widespread expression of CD47 on peripheral cells raises concerns regarding off-tumor and off-CNS toxicities, such as anemia and thrombocytopenia, which have hindered the efficacy of anti-CD47 agents in oncology (Zhao et al. 2022; Pei et al. 2025). The relative CNS selectivity of SIGLEC10 expression may provide a safety benefit for ONC-841, although its efficacy has yet to be clinically validated.

## Advanced immunotherapies for protein aggregate clearance

Expanding on the potential of central NLRP3 inhibition to mitigate upstream neuroinflammatory triggers, a concurrent and equally revolutionary trend of innovation is directed towards the precise elimination of the pathological protein aggregates characteristic of neurodegenerative diseases. Conventional monoclonal antibodies (mAbs) targeting Aβ have shown clinical proof-of-concept for immunotherapy in AD; however, their effectiveness has been limited by challenges such as insufficient brain penetration, transient activity, and the necessity for extended, high-dose treatment. This section examines advanced immunotherapies that address these limitations by targeting innovative immune checkpoints to restore intrinsic microglial function and by developing resilient, cell-based therapies capable of programmable, aggregate-selective clearance (Cabral et al. 2025; Fu et al. 2025; Pesce et al. 2026).

### Programmable CAR-based immunotherapies: precision cell engineering against proteinopathies

Although antibodies such as ONC-841 provide a systemic method, the most groundbreaking progress in aggregate clearance is derived from the remarkable achievements of chimeric antigen receptor (CAR) T cells in cancer treatment (Fieldhouse 2026). Researchers are currently developing CAR immunotherapies for CNS proteinopathies to overcome the inherent limitations of traditional antibodies: limited brain penetration, brief half-life, and reliance on passive immunization (Pesce et al. 2026). CAR-based therapies entail the modification of a patient’s immune cells (T cells, or potentially macrophages or NK cells) to express a synthetic receptor that guides them to a designated target. This technology is being modified to identify pathological protein conformations in neurodegenerative diseases (Katsaros and Mougiakakos 2025).

CAR-based therapies offer durable, programmable aggregate clearance with single dosing, overcoming the limited CNS penetration and short half-life of conventional antibodies. A recent comprehensive reviews outline the design principles for next-generation therapies, highlighting the imperative of "tunable controls" to guarantee that the potent cytotoxic mechanisms of CAR-T cells align with the CNS’s limited tolerance for inflammation (Pesce et al. 2026; Garcia-Robledo et al. 2026; Zhang et al. 2026). This signifies a transition from passive immunotherapy to active cellular immunotherapy for the clearance of protein aggregates.

### Composite biomarkers for selecting patients and confirming target engagement

The successful clinical deployment of these innovative immunotherapies hinges on our ability to select the right patients and demonstrate on-target engagement (Eijsvogel et al. 2024; Garg et al. 2024). A major recent advance is the development of fluid biomarkers that simultaneously capture both the protein aggregation pathology and the neuroinflammatory response (Heneka et al. 2025; Valletta et al. 2025).

**Fig 4:**
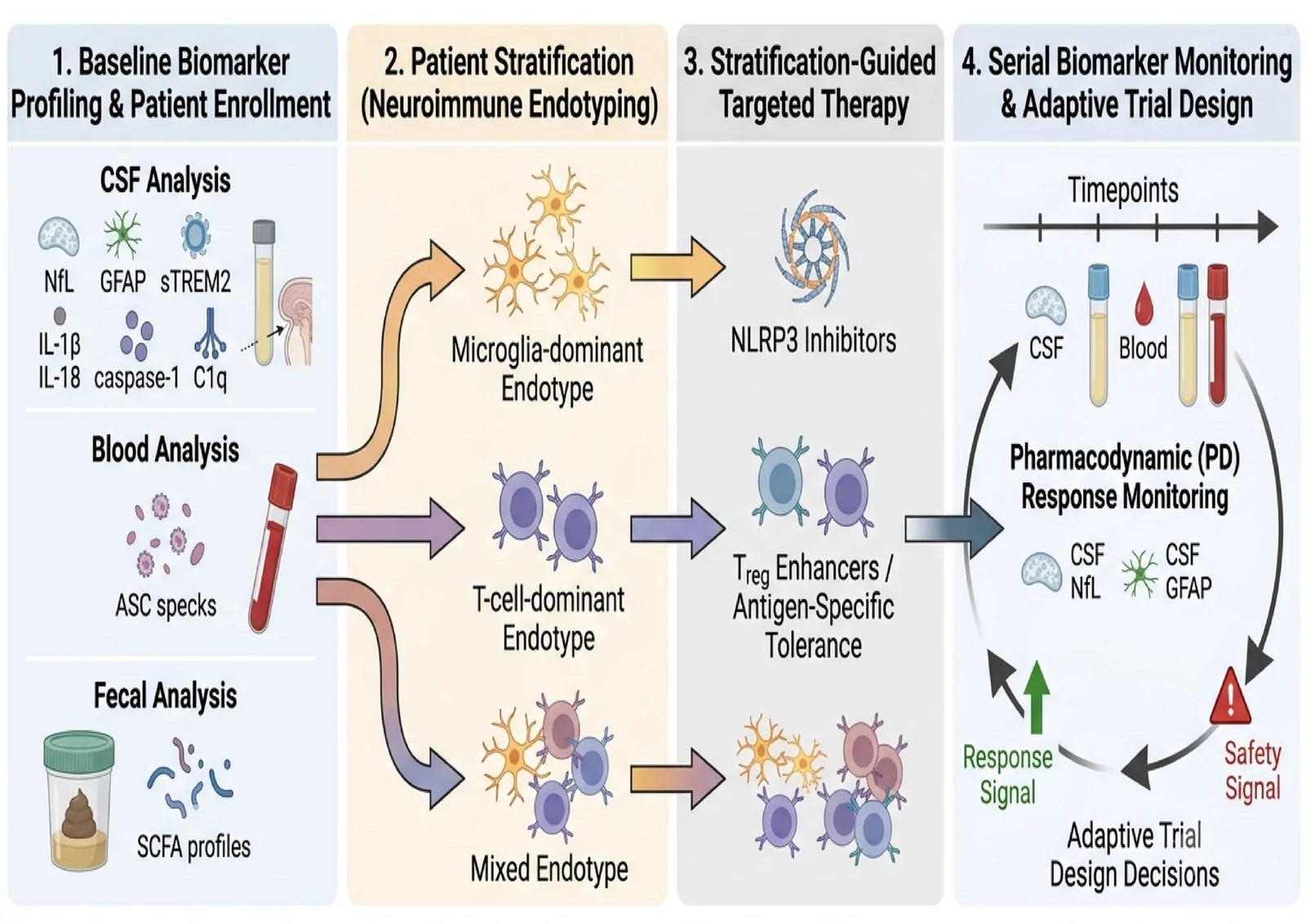
A roadmap for precision neuroimmunology in Alzheimer’s and Parkinson’s diseases. This schematic illustrates the proposed biomarker-guided approach for patient stratification and treatment monitoring in neuroimmune-targeted clinical trials. Baseline biomarker profiling (CSF analysis for NfL, GFAP, sTREM2, IL-1β, IL-18, caspase-1, C1q; blood analysis for ASC specks; fecal analysis for SCFA profiles) enables assignment of patients to neuroimmune endotypes (microglia-dominant, T-cell-dominant, or mixed endotype). This stratification guides targeted therapy selection (e.g., NLRP3 inhibitors for microglia-dominant and Treg enhancers or antigen-specific tolerance for T-cell-dominant). Serial biomarker monitoring (including CSF NfL and GFAP for pharmacodynamic responses) allows adaptive trial design and early detection of response or safety signals

Lobanova et al. (2024a and b) established an ultra-sensitive single-molecule assay to measure ASC specks, oligomeric assemblies of the inflammasome adaptor protein ASC, in human biofluids. These ASC specks are released during the activation of the microglial inflammasome and function as extracellular signals that amplify inflammation. The research indica ted an increased quantity of ASC specks in the bloodstream of patients with early-stage AD and PD (Lobanova et al. 2024a). A composite biomarker that integrates ASC speck counts with levels of pathological proteins (pTau or α-synuclein) normalized against Aβ can effectively differentiate early-stage patients from healthy controls, achieving accuracies of 92% for AD and 97% for PD(Lobanova et al. 2024a; Clayton et al. 2026) (Fig. 4).

Such composite biomarkers are invaluable for effectively stratifying patients in clinical trials of anti-inflammatory or aggregate-clearing therapies (Rao et al. 2026; Raj et al. 2025; Dessing et al. 2025). They provide pharmacodynamic evidence that a drug, such as ONC-841 or a CAR-T cell, is effectively engaging its target and modulating the neuroimmune environment. Additionally, these biomarkers enable the longitudinal monitoring of treatment responses through a simple blood test(Li et al. 2024; Levstek et al. 2024) (Table 3).

**Table 3:** Proposed composite biomarker panel for neuroimmune trials

Biomarker category	Biomarker	Evidence level	Biofluid	What It measures	Clinical utility	Refs
Target engagement	IL-1β, IL-18	Exploratory/research-stage	CSF	NLRP3 inflammasome activity	Confirm drug hits target (e.g., NT-0796)	Clarke et al. 2025; Doedens et al. 2024)
Target engagement	Caspase-1 activity	Exploratory/research-stage	CSF	Proximal inflammasome function	Confirm NLRP3 inhibition	Gong et al. 2022; Taylor et al. 2024; Adamczak et al. 2012)
Pharmacodynamic	Neurofilament light chain (NfL)	Clinically validated (Class II)	CSF	Axonal injury	Monitor neuroprotection (response to therapy)	Machacek et al. 2024; Pawlitzki et al. 2019; Bergman et al. 2016; Villar et al. 2015)
Pharmacodynamic	glial fibrillary acidic protein (GFAP)	Clinically validated (Class II)	CSF	Reactive astrogliosis	Monitor glial response	Evertsson et al. 2025; Youn et al. 2025; Cetindag et al. 2026; Pelkmans et al. 2024)
Pharmacodynamic	sTREM2	Emerging with replicated evidence (Class III)	CSF	Microglial activation state	Monitor ONC-841 response	Dong et al. 2022; Ewers et al. 2020; Tuil et al. 2025; Suárez‐Calvet et al. 2016; Wood 2017)
Pharmacodynamic	ASC specks	Exploratory/research-stage	Blood	Inflammasome-derived extracellular signals	Non-invasive serial monitoring	Topping et al. 2025; Lobanova et al. 2024b)
Predictive	C1q (baseline)	Emerging with replicated evidence (Class III)	CSF	Pre-existing complement activation	Enrich for ANX005 responders	Mohammad et al. 2025; Kumar et al. 2026; Zhang et al. 2023c; Tortosa-Carreres et al. 2024; Håkansson et al. 2020)
Predictive	T cell clonality	Exploratory/research-stage	CSF	Adaptive immune infiltration	Stratify T-cell-dominant endotypes	Gate et al. 2020b; Hayashi et al. 2026; Pappalardo et al. 2020)
Predictive	SCFA profile	Exploratory/research-stage	Feces	Gut microbiome status	Stratify for microbiome interventions	Oliver et al. 2024; Ahmad et al. 2025; Siddiqui et al. 2017; Nogal et al. 2023; Quinn-Bohmann et al. 2024)

## Limitations and future directions

The previous sections have outlined a varied and swiftly advancing array of therapeutic strategies aimed at specific components of the integrated neuroimmune network, including NLRP3 inflammasome inhibitors, complement blockers, microglial checkpoint modulators, peripheral signaling hub interventions, and CAR-based immunotherapies (Table 4).

**Table 4:** Overview of investigational therapeutic strategies targeting the neuroimmune network in Alzheimer’s and Parkinson’s diseases: developmental status, key advantages, and limitations

Target node	Therapeutic strategy	Example agent(s)	Developmental stage	Regulatory status	Key advantage	Key limitation	Refs
Microglial inflammasome	NLRP3 inhibition	NT-0796, MCC950 (discontinued)	Phase 1b/2a (PD)	**Investigational**	Central node; broad anti-inflammatory effect	MCC950 hepatotoxicity; long-term safety unknown	Clarke et al. 2025; Chu et al. 2026)
Complement cascade	C1q inhibition	ANX005	Phase 2/3 (HD, GBS)	**Investigational**	Preserves synaptic integrity	Only benefits C1q-high subgroup	Mohammad et al. 2025; Kumar et al. 2022; Lansita et al. 2017)
Complement cascade	C5aR1 antagonism	PMX53/205	Preclinical (AD)	**Investigational**	Oral, CNS-penetrant	Preclinical only	Gomez-Arboledas et al. 2022; Kumar et al. 2020)
Microglial checkpoint	SIGLEC10 blockade	ONC-841	Preclinical (AD)	**Investigational; not yet in human trials**	Restores phagocytosis of Aβ and tau	Translation to humans unproven	Wang et al. 2025a)
Microglial checkpoint	CD47-SIRPα blockade	AO-176	Preclinical	**Investigational**	Enhances phagocytosis	Off-target anemia risk	Puro et al. 2020; Maute et al. 2022)
Adaptive immunity (T cells)	Treg expansion	Low-dose IL-2	Phase I (AD)	**Investigational**	Suppresses effector T cells	Requires chronic administration	Faridar et al. 2025)
Adaptive immunity (T cells)	Antigen-specific tolerance	Tolerogenic vaccines (α-synuclein, tau) ACI-7104.056	Preclinical; Phase 2 ongoing	**Investigational**	Precision; avoids global immunosuppression	Antigens not fully defined	Faas et al. 2025b; Moorman et al. 2021)
Peripheral signaling	IL-20 family neutralization	Anti-IL-20 mAbs	Preclinical (repurposed)	**Investigational**	Targets upstream peripheral signals	Redundancy with other cytokines	Kragstrup et al. 2018; Maggisano et al. 2025)
Peripheral signaling	Microbiome modulation	FMT, C. butyricum	Preclinical/Exploratory (PD)	**Investigational**	Restores homeostatic signaling	Highly variable between individuals	Sun et al. 2021; Wang et al. 2023)
Protein aggregate clearance	CAR-T / CAR-NK cells	Anti-Aβ, anti-tau CARs	Preclinical	**Investigational**	Durable, single infusion; programmable	CNS delivery; CRS risk; unproven in humans	Boskovic et al. 2026)
Protein aggregate clearance	Passive immunization	Anti-Aβ mAbs (lecanemab, donanemab)	Approved (AD)	**FDA-approved**	Clinically validated mechanism	Modest efficacy; ARIA; poor CNS penetration	Avgerinos et al. 2024; Alkhalifa et al. 2025)

While this pipeline represents remarkable progress, several conceptual, technical, and translational limitations cut across multiple strategies and must be acknowledged. Addressing these shared gaps will define the next decade of neuroimmune drug development. The bolded regulatory status emphasizes the current approval or investigational status of the primary agent (e.g., Investigational for NT-0796, FDA-approved for anti-Aβ mAbs).

### Current limitations

The translation of neuroimmune therapies for neurodegenerative diseases faces major challenges. The integrated neuroimmune network exhibits considerable redundancy, such that inhibiting a single node like the NLRP3 inflammasome may be offset by upregulation of parallel pathways (e.g., TLR4 or NLRC4). safety uncertainties for chronic CNS immunomodulation persist: first-in-class trials (e.g., NT-0796, ANX005, ONC-841) report acceptable short-term safety, but long-term consequences, including susceptibility to CNS infections, impaired synaptic pruning essential for memory consolidation, and accelerated protein aggregation due to disrupted microglial surveillance, remain unknown. The heterogeneity of neuroimmune endotypes means that patients with AD or PD are not uniform; some exhibit microglial-dominant activation while others show T-cell-dominant inflammation, yet most trials enroll all-comers, diluting treatment effects, and biomarker-based stratification remains the exception.

Biomarker development lags behind therapeutic innovation. While CSF markers (NfL, GFAP, sTREM2) show reasonable evidence for monitoring pharmacodynamic responses, the predictive utility of novel candidates, including IL-19, IL-20, IL-22, and T-cell clonality, remains exploratory. The field requires large-scale, multi-center validation studies to establish the sensitivity, specificity, and clinical utility of these markers before they can be incorporated into routine trial design or clinical practice.

To enhance precision neuroimmunology, adaptive platform trials ought to evaluate multi-node or sequential strategies.

### Biomarker validation gaps

It is important to acknowledge that the proposed composite biomarker framework reflects a conceptual roadmap rather than a validated clinical tool, as the level of evidence supporting each candidate marker varies considerably. NfL and GFAP have achieved a degree of clinical validation, with well-established analytical performance, replicated findings across large cohorts, and integration into clinical trial designs and diagnostic frameworks. In contrast, the majority of neuroimmune biomarkers discussed in this review, including sTREM2, ASC specks, T-cell clonality, caspase-1 activity, IL-1β, IL-18, and SCFA profiles, remain at an exploratory or research-stage level, supported by promising but limited evidence from small cohorts or early-phase trials. These candidates have not undergone the rigorous analytical validation, large-scale prospective replication, and regulatory qualification required for routine clinical deployment.

Consequently, while these biomarkers hold considerable mechanistic and translational promise, their current use should be confined to research contexts with appropriate caution, and their inclusion in clinical trials should be accompanied by prespecified statistical plans and independent replication strategies. The field urgently needs coordinated efforts to standardize assays, conduct multi-center validation studies, and obtain regulatory approval to close the gap between discovery and clinical use. This will ensure that future neuroimmune therapies are associated with robust biomarkers that can reliably facilitate patient selection, validate target engagement, and monitor treatment response.

### Future directions

To enhance precision neuroimmunology, adaptive platform trials ought to evaluate multi-node or sequential strategies, including the combination of NLRP3 inhibitors with Treg enhancers, the sequencing of SIGLEC10 blockade followed by IL-20 family blockers, or the induction of antigen-specific tolerance prior to CAR-T administration. The regulatory qualification of a composite biomarker panel is urgently required, encompassing CSF markers (NfL, GFAP, IL-1β, IL-18, caspase-1, C1q, sC5b-9, T cell clonality, and IFN-γ), blood ASC specks, and next-generation PET ligands for P2Y12 and CSF1R. Furthermore, preclinical models should transition to aged (≥ 18 months) humanized mice that express human SIGLEC10, TREM2 variants, or the IL-20 receptor complex, incorporating chronic dosing and co-morbidities.

Thorough multi-omics endotyping (single-cell RNA/ATAC-seq of cerebrospinal fluid, plasma/cerebrospinal fluid proteomics, and gut metagenomics) will delineate neuroimmune endotypes such as “microglia-high/Treg-low” to facilitate basket trial allocation. All phase 3 trials and sanctioned therapies must include mandatory long-term safety registries (≥ 5 years) that monitor CNS infections, autoimmune encephalitis, and cognitive deterioration. Cellular immunotherapies administered via the central nervous system, including intrathecal/intracerebroventricular CAR-T or CAR-macrophages, off-the-shelf allogeneic CAR-NK cells, and safety mechanisms such as inducible caspase-9, require thorough examination to address delivery and toxicity challenges.

## Conclusion

The therapeutic approach for neurodegenerative disorders is evolving from a focus on neurons and microglia to a comprehensive neuroimmune network model that acknowledges the intricate interactions among CNS immune cells, invading adaptive lymphocytes, and external signaling centers. This paradigm transformation is currently being implemented in clinical advancements, as illustrated by pioneering investigational compounds such as the NLRP3 inhibitor NT-0796, which infiltrates the CNS (showing reductions in biomarkers related to axonal injury and T-cell activation in Parkinson’s disease), the anti-SIGLEC10 antibody ONC-841 (boosting microglial phagocytosis of protein aggregates), and novel CAR-based immunotherapies that present the possibility of sustained aggregate elimination. These methodologies collectively signify a transformative change from passive immunosuppression to proactive immunomodulation, deliberately focusing on particular points within the neuroimmune system.

Clinical translation faces many challenges, such as network redundancy that limits the effectiveness of monotherapies, slow progress in biomarker development relative to therapeutic innovation and persistent long-term safety concerns. Further strategies are likely to involve multi-node integrations, e.g., NLRP3 inhibition in combination with Treg enhancement, and the proposed triadic biomarker framework (target engagement, pharmacodynamic responses, and predictive endotyping) provides a roadmap for patient classification, although rigorous validation on a large scale is required.

Adaptive platform trials employing sequential or combinatorial approaches, along with mandatory long-term safety registries, will be critical for establishing efficacy and safety. The best hope for the development of DMTs that can significantly change the course of AD, PD, and related neurodegenerative diseases is to accept the intricacies of the neuroimmune network rather than to look for simplistic solutions in the end.

## Data Availability

No data was used for the research described in the article.

## Abbreviations

AAT: Alpha-1-antitrypsin
AD: Alzheimer’s disease
ADX-914: CNS-penetrant C5aR1 antagonist
AMPK: AMP-activated protein kinase
ANX005: Anti-C1q monoclonal antibody
AO-176: Anti-CD47 antibody
ARIA: Amyloid-related imaging abnormalities
ASC: Apoptosis-associated speck-like protein containing a CARD
Aβ: Amyloid-beta
BAM: Border-associated macrophage
BBB: Blood-brain barrier
C1q: Complement component 1q
C5aR1: Complement component 5a receptor 1
CAR: Chimeric antigen receptor
CCR2/4: C-C chemokine receptor type 2/4
CD24: Cluster of differentiation 24
CD33: Cluster of differentiation 33
CD47: Cluster of differentiation 47
CD8⁺: Cluster of differentiation 8 positive
CD163: Cluster of differentiation 163
CD200/R: Cluster of differentiation 200 / receptor
CNS: Central nervous system
CSF: Cerebrospinal fluid
CSF1R: Colony-stimulating factor 1 receptor
CXCL10: C-X-C motif chemokine ligand 10
DAM: Disease-associated microglia
FGF21: Fibroblast growth factor 21
FTD: Frontotemporal dementia
GFAP: Glial fibrillary acidic protein
HD: Huntington’s disease
IFN-γ: Interferon-gamma
Th1/Th17: T helper type 1/17
TLR4: Toll-like receptor 4
TNF-α: Tumor necrosis factor-alpha
VLA-4: Very late antigen-4 (integrin α4β1)
IL-1β: Interleukin-1 beta
IL-2: Interleukin-2
IL-6: Interleukin-6
IL-17: Interleukin-17
IL-18: Interleukin-18
IL-19: Interleukin-19
IL-20: Interleukin-20
IL-20R1/R2: Interleukin-20 receptor subunit 1/2
IL-22: Interleukin-22
IL-24: Interleukin-24
IPA: Indole-3-propionic acid
mAbs: Monoclonal antibodies
MHC-I/II: Major histocompatibility complex class I/II
MS: Multiple sclerosis
NF-κB: Nuclear factor kappa-light-chain-enhancer of activated B cells
NfL: Neurofilament light chain
NK cell: Natural killer cell
NLRP3: NACHT, LRR, and PYD domains-containing protein 3
NSAID: Non-steroidal anti-inflammatory drug
NT-0796: CNS-penetrant NLRP3 inhibitor
ONC-841: Anti-SIGLEC10 monoclonal antibody
PD: Parkinson’s disease
PD-1/PD-L1: Programmed cell death protein 1 / ligand 1
PET: Positron emission tomography
PMX53/205: C5aR1 antagonist
pTau: Hyperphosphorylated tau
SCFA: Short-chain fatty acid
SIGLEC10: Sialic acid-binding Ig-like lectin 10
SIRPα: Signal regulatory protein alpha
sTREM2: Soluble triggering receptor expressed on myeloid cells 2
TCR: T cell receptor
TREM2: Triggering receptor expressed on myeloid cells 2
Treg: Regulatory T cell
TSPO: Translocator protein
